# Comparative Outcomes of Gross Total Resection vs. Subtotal Resection Plus Radiotherapy for Preventing Craniopharyngioma Recurrence: A Meta-Analysis of the Endoscopic Endonasal Approach

**DOI:** 10.3390/cancers17152516

**Published:** 2025-07-30

**Authors:** Ernest J. Bobeff, Bartosz Szmyd, Wojciech Młynarski, Emmanuel Jouanneau, Caroline Apra, Ming Shen, Zara M. Patel, Dariusz J. Jaskólski, Theodore H. Schwartz

**Affiliations:** 1 Department of Neurosurgery and Neuro-Oncology, Medical University of Lodz, Barlicki University Hospital, Kopcinskiego St. 22, 90-153 Lodz, Poland; bartoszmyd@gmail.com (B.S.); dariusz.jaskolski@umed.lodz.pl (D.J.J.); 2 Department of Sleep Medicine and Metabolic Disorders, Medical University of Lodz, Mazowieka St. 6/8, 92-251 Lodz, Poland; 3 Department of Pediatrics, Oncology and Hematology, Medical University of Lodz, Sporna St. 36/50, 91-738 Lodz, Poland; wojciech.mlynarski@umed.lodz.pl; 4 Skull Base and Pituitary Surgical Department, Neurological Hospital Pierre Wertheimer, Hospices Civils de Lyon, 69002 Lyon, France; emmanuel.jouanneau@chu-lyon.fr; 5 Inserm U1052, CNRS UMR5286, Lyon I University, Signaling, Metabolism and Tumor Progression, Cancer Center of Lyon, 69373 Lyon, France; 6 Neurosurgery Department, Henri Mondor University Hospital, 94000 Créteil, France; caroline.apra@aphp.fr; 7 Biotherapies Group, INSERM U955, Mondor Biomedical Research Institute, 94010 Créteil, France; 8 Neurosurgery Department, Henri Mondor Hospital, Assistance Publique-Hôpitaux de Paris, 94010 Créteil, France; 9 Shanghai Neurosurgical Center, Department of Neurosurgery, Huashan Hospital, Shanghai Medical School, Fudan University, 958#, Jinguang Road, Shanghai 200040, China; 062105346@fudan.edu.cn; 10 Division of Rhinology, Department of Otolaryngology-Head and Neck Surgery, Stanford University School of Medicine, Palo Alto, CA 94304, USA; zmpatel@stanford.edu; 11 Independent Researcher, New York, NY, USA

**Keywords:** craniopharyngioma, GTR, STR+XRT, progression, outcome

## Abstract

Objective: Craniopharyngioma recurrence risk studies comparing gross total resection (GTR) vs. subtotal resection (STR) with radiotherapy (XRT) provide inconclusive or contradictory results. This may be an effect of the small group sizes and diversity in the approaches used. Currently, the endoscopic endonasal approach (EEA) is preferred in craniopharyngioma management. Here, we aimed to perform a meta-analysis comparing recurrence risk after GTR vs. STR plus XRT in patients treated with the EEA regimen. Methods: We performed a systematic literature search of original English language papers on craniopharyngioma management published in the PubMed, Web of Science, and Scopus databases up to 18 October 2023. Eleven articles included data on recurrence rate after EEA: GTR vs. STR with XRT. We extracted the year of publication, number of patients, surgical approach, extent of resection, and follow-up duration. We used meta-analysis for the odds ratio (OR) in fixed and random effects models and Egger’s and Begg’s tests to assess heterogeneity and publication bias. Follow-up duration and time to recurrence were additionally included in Kaplan–Meier curves with log-rank test analysis. Results: We observed a lower recurrence rate in patients after GTR (10%) as compared to STR with XRT (30%), OR = 0.299, *p* < 0.001. To increase data reliability, we limited our analysis to studies with at least five patients in each subgroup and also observed lower recurrence in patients after GTR (12%) as compared to STR with XRT (27%), OR = 0.376, *p* = 0.011. Survival analysis confirmed significant differences in recurrence-free survival percentages between these groups (*p* = 0.008). Conclusions: To date, this is the largest meta-analysis evaluating the recurrence risk in patients undergoing EEA for craniopharyngioma resection, comparing outcomes between those treated with GTR and those treated with STR plus XRT. The results suggest that GTR significantly reduces recurrence risk.

## Simple Summary

Craniopharyngioma recurrence rates after surgery remain debated, especially when comparing gross total resection (GTR) with subtotal resection (STR) followed by radiotherapy (XRT). This meta-analysis focused on patients treated with the modern endoscopic endonasal approach (EEA). Data from 11 studies were analyzed, including recurrence rates, surgical method, and follow-up. Results showed a significantly lower recurrence rate after GTR (10%) compared to STR with XRT (30%; OR = 0.299, *p* < 0.001). This is the largest meta-analysis to date on EEA-treated craniopharyngiomas, supporting GTR as the more effective option in reducing recurrence risk.

## 1. Introduction

Craniopharyngiomas, though histologically benign, pose significant challenges in both pediatric and adult populations due to their high recurrence rate [1,2,3]. In children, these tumors are typically adamantinomatous, while in adults, they may also be of the papillary subtype [4,5]. The former are associated with CTNNB1 mutations, while the latter are linked to BRAF V600E mutations [6,7,8,9,10,11].

Craniopharyngiomas arise in the hypothalamic–pituitary region, frequently involving the pituitary stalk [12]. Surgery is generally first-line therapy with the goal of relieving mass effect in the case of visual loss or hydrocephalus, obtaining diagnostic tissue, since the papillary subtype may be amenable to chemotherapy, and, in some cases, to achieve a gross total resection (GTR) to achieve a durable cure [13]. Since GTR can lead to pituitary–hypothalamic morbidity, some authors advocate for subtotal resection (STR) followed by radiotherapy (XRT), and some studies have shown that these two treatments may be equally effective at preventing tumor recurrence [13,14,15,16,17,18,19,20,21,22,23]. Ultimately, the goal of treatment is to prevent recurrence, which still occurs in more than 50% of cases [1,2,3,12,24,25,26,27,28,29,30]. Despite recurrences, long-term survival rates are high, with 10- and 20-year overall survival exceeding 50% [31].

Earlier studies that showed equipoise for GTR versus STR and radiation were mostly conducted in an era where craniopharyngiomas were treated with a craniotomy. More recently, these tumors have been removed through the endoscopic endonasal approach (EEA), which provides better visualization of the tumor and its relationship to surrounding normal neurovascular structures, thereby providing the surgeon a greater ability to achieve GTR with lower morbidity [32]. While the morbidity of EEA has been well-described in several prior reports, there is little data comparing recurrence rates following GTR alone versus STR plus radiation, particularly when the surgery has been performed using EEA and not a craniotomy. We hypothesized that the unique perspective provided by EEA would result in higher tumor control rates following GTR, so we performed a systematic review of the existing literature to address this question.

## 2. Materials and Methods

The current meta-analysis was performed in concordance with the Preferred Reporting Items for Systematic Reviews and Meta-Analyses (PRISMA) guidelines. We double-screened (E.J.B. and B.M.Sz) all the relevant original English language papers published in the PubMed, Web of Science, and Scopus databases before 18 October 2023. In the case of any discrepancies between the two authors extracting data, the final decision was made by the senior authors (D.J.J. and T.H.S.).

**Figure 1:**
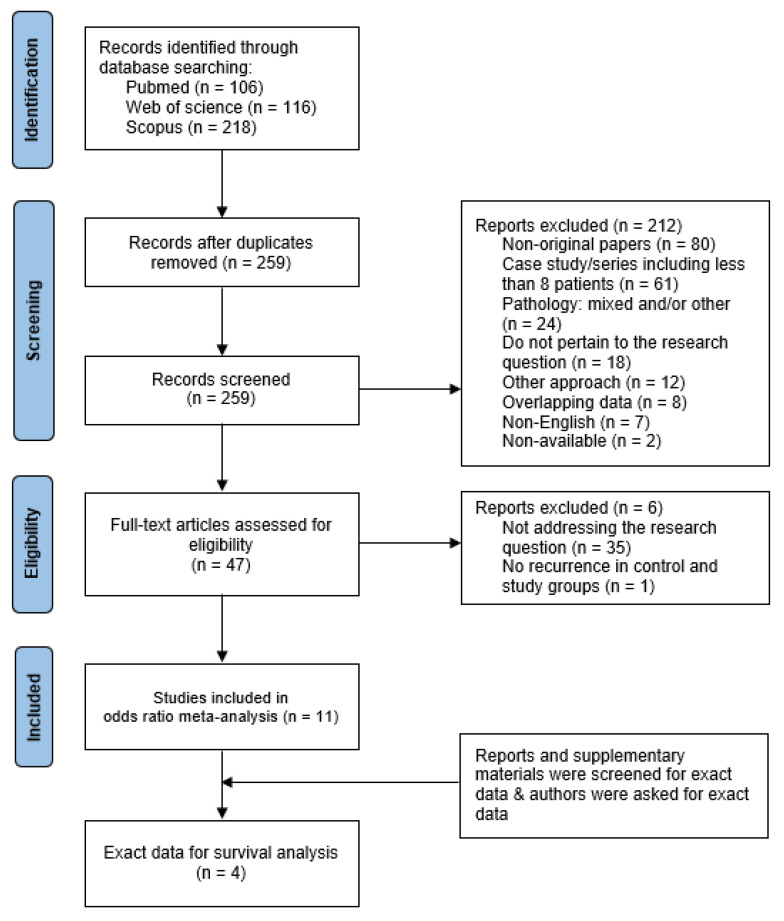
The PRISMA flowchart for assessing the link between gross total resection vs. subtotal resection with radiation therapy and the recurrence risk of craniopharyngioma after endoscopic endonasal approach resection.

We used the following query: (“craniopharyngioma”) AND (“radiotherapy” OR “radiation” OR “radiosurgery”) AND (“endoscopic” OR “endoscope” OR “endoscopy”). The eligibility criteria were (1) a diagnosis of craniopharyngioma, (2) EEA resection, (3) information about radiation, and (4) information about recurrence. We took both recurrence and progression into account, but for simplicity, we referred to both as “recurrence”; in the search strategy, a total of 440 papers were identified, totaling 259 articles after eliminating duplicates. These records were double-screened. In total, 47 original papers potentially pertaining to the research topic were enrolled in full-text assessment for eligibility (see Figure 1). Eleven of these were included in the meta-analysis [13,14,15,16,17,18,19,20,21,22,23].

Data Extraction: Data were extracted manually by two of us (E.J.B. and B.M.Sz) using a standardized form encompassing the year of publication, total number of patients, number of children/adults, extent of resection, use of XRT, duration of follow-up, and recurrence/no recurrence.

Statistical Analysis: We performed a meta-analysis of the odds ratio (OR), with an assessment of heterogeneity and publication bias (Egger’s and Begg’s tests) performed using MedCalc Statistical Software version 19.1.2 (MedCalc Software, Ostend, Belgium). The impact of follow-up time and time to recurrence on its risk was taken into consideration in survival analysis with the usage of the Kaplan–Meier curve with the log-rank test. This step was performed using GraphPad Prism 8.2 (Prisma, San Diego, CA, USA).

## 3. Results

### 3.1. Meta-Analysis

We obtained 11 studies that met the inclusion criteria, published between 2013 and 2023 (see Table 1). The group consisted of 431 patients after EEA for craniopharyngioma resection. The mean/median follow-up time varied from 2 to 8.6 years, after which the overall recurrence rate was 14%.

**Figure 2:**
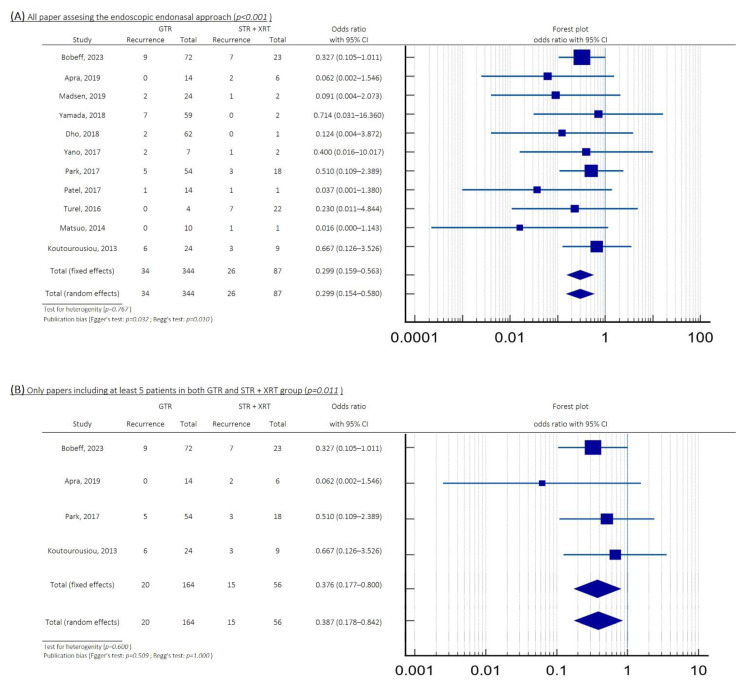
Forest plots showing the comparison between GTR and STR plus XRT groups in patients after EEA for craniopharyngioma resection: (**A**) all included studies, OR = 0.299 (95%CI: 0.159–0.563; *p* < 0.001); (**B**) studies with at least 5 patients in each subgroup, OR = 0.376 (95%CI: 0.177–0.800; *p* = 0.011). Abbreviations: CI, confidence interval; EEA, endoscopic endonasal approach; GTR, gross total resection; OR, odds ratio; XRT, radiation therapy; STR, subtotal resection.

Regarding all 11 studies, the risk of recurrence was significantly lower in the 344 patients who underwent GTR (10%) compared to the 87 patients who received STR with XRT (30%), with an OR = 0.299 (95%CI: 0.159–0.563; *p* < 0.001; see Figure 2A). The test for heterogeneity revealed *p* = 0.767, while publication bias tests showed *p* = 0.032 (Egger’s) and *p* = 0.010 (Begg’s test). To increase data reliability, we limited our analysis to studies with at least five patients in each subgroup. Recurrence rates were significantly lower after GTR (12%) compared to STR plus XRT (27%), with an OR = 0.367 (95%CI: 0.177–0.800; *p* = 0.011; see Figure 2B). The test for heterogeneity revealed *p* = 0.600, while publication bias tests showed *p* = 0.509 (Egger’s) and *p* = 1.000 (Begg’s test).

### 3.2. Survival Analysis

**Figure 3:**
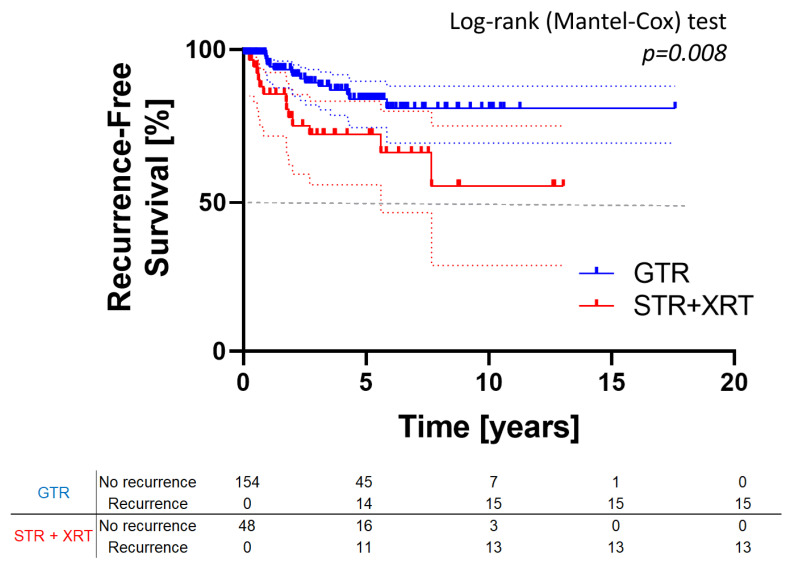
Kaplan–Meier curve assessing the recurrence-free survival in patients treated using EEA between GTR and STR plus XRT subgroups (*p* = 0.008). Abbreviations: EEA, endoscopic endonasal approach; GTR, gross total resection; OR, odds ratio; XRT, radiation therapy; STR, subtotal resection.

We obtained raw data, including time to recurrence and follow-up duration, from four publications [13,15,16,17], all published between 2017 and 2023. These studies encompass a total of 202 patients, with 154 treated via GTR and 48 with STR plus XRT (see Table 2). Survival analysis (Kaplan–Meier curve with log-rank test) confirmed previous findings, showing a significantly lower recurrence risk in patients treated with GTR compared to STR+XRT (*p* = 0.008; see Figure 3).

## 4. Discussion

We present the largest meta-analysis of recurrence risk in patients treated with EEA. Our analysis showed that GTR reduces recurrence risk by 20% compared with STR and XRT. Time-to-recurrence data and Kaplan–Meier analysis further support the superiority of GTR in prolonging recurrence-free survival. By including only studies with at least five patients per subgroup, we strengthened the reliability of our findings, showing significantly lower recurrence rates in the GTR group (12%) compared to the STR with XRT group (27%).

Treating craniopharyngiomas presents a clinical challenge that requires careful balance between tumor control and the risk of complications, such as damage to the hypothalamus and pituitary gland [33,34]. Adjuvant therapy such as XRT or chemotherapy is often recommended if GTR cannot be achieved [35], and some authors claim that it is equivalent to GTR [1]. However, more recent studies in which EEA was performed suggest that it may not be as effective. For example, Apra et al. (2019) initially favored STR with XRT, but observed faster tumor recurrences, more hypothalamic invasion, and more severe symptoms, using this strategy [17]. This led them to pursue GTR, even in cases where the tumor compressed the hypothalamus. The rationale for this approach was that less aggressive resections in such cases still did not prevent hypothalamic damage. They also found that preserving the pituitary stalk often does not prevent panhypopituitarism and DI, and also leads to a higher recurrence rate.

A very high GTR rate (91%) has been reported by Dho et al., especially in first-time surgeries, regardless of the tumor size [21]. Importantly, the authors found that performing GTR did not increase the complication rate. STR often involved only small tumor remnants adhered to the basilar artery, internal carotid artery, hypothalamus, or a cystic portion invading the frontal lobe, where complete removal of the cyst wall was not possible [21]. Another study recommended GTR whenever possible. In children (aged 4–18 years), they tend to be even more aggressive, with roughly half undergoing GTR and the other half achieving near-total resection (NTR > 95%), as there are strict restrictions regarding radiotherapy in this age group [18].

Several authors have tried to create anatomic classifications that might relate to the ability to achieve GTR. Park et al. (2017) observed the lowest rate of GTR in retroinfundibular tumors (22%), compared to other types (48%), although this difference was not statistically significant [16]. Dho et al. (2018) noted the lowest GTR rate in retrochiasmatic tumors with an incompetent diaphragma sellae (93%), although GTR rates in general were high in their series [21]. Similarly, Patel et al. (2017) reported a very high GTR rate (94%); in one case, however, they opted for STR due to extensive tumor spread into the carotid canals [15]. Yano et al. (2017) used Puget’s classification, which categorizes craniopharyngiomas preoperatively based on hypothalamic compression and is generally related to tumor size: grade 0 (no involvement), grade 1 (displacement), and grade 2 (clear invasion) [14]. Postoperatively, Puget’s grading assesses hypothalamic damage: grade 0 (no symptoms), grade 1 (minimal damage), and grade 2 (severe damage). The authors noted that only one patient showed an increase in Puget’s grading, while GTR in all cases allowed for a reduction in Puget’s grading by one or even two levels [14]. This highlights the safety of GTR when performed in experienced centers. In our previous study, we used the QST classification, which encompasses craniopharyngiomas within the sella turcica (type Q), those growing in the third ventricle (type T), and intermediate cases where the pituitary stalk is not visible (type S). So far, we have not observed a clear association between tumor type and the extent of resection [13].

Ideally, studies on craniopharyngiomas should separate the population into children and adults. Since there are very few EEA studies that specifically describe the relationship between the extent of resection and recurrence, we combined all age groups. Some studies focus exclusively on children [15,20,22], while others address only adults [17,19,21]. Additionally, some studies consider a mixed population [13,14,16,18]. Without sharing raw data in accessible online databases, these studies were ineligible for subgroup meta-analysis. Furthermore, authors use varying age cutoffs, at 18 years [13,15,16,17,18,19,20], 17 years [22], and 15 years [21], which makes it difficult to combine studies. Among adults, this difference was considerably smaller, with recurrence rates of 11.5% following GTR and 19.4% after STR with XRT (Table 2). These findings may inform treatment selection and underscore the importance separating pediatric and adult populations.

### Study Limitations

There are several limitations to this paper. A primary challenge we faced was the accurate and comprehensive extraction of data from texts. There were, in some instances, missing data concerning age, sex, histopathological type of craniopharyngioma, and follow-up duration. The accumulation of such minor issues across multiple studies may potentially lead to faulty conclusions [36]. Since there were limited papers published on the topic, we had to combine adult and pediatric cases and also combine recurrent and newly diagnosed cases. It is possible that our conclusions do not hold for all of these subgroups. This paper is based on a review of retrospective studies and is prone to the inherent bias of such studies that often depend on ambiguous criteria for case selection.

Additionally, a significant limitation of our study is the lack of consistent and comprehensive data on key clinical outcomes beyond recurrence, such as endocrine function, visual improvement, overall survival, and quality of life. This limited the scope of our analysis and prevented a more holistic comparison of treatment strategies. A further methodological limitation of our study is the inability to control for factors influencing the choice and outcome of treatment strategy—such as tumor location, size, center experience, or patient preferences—which may bias comparisons between GTR and STR+RTx in retrospective data. Additionally, the extent of resection may reflect not only preoperative planning but also intraoperative circumstances, further complicating interpretation.

Moreover, a formal critical appraisal of the risk of bias in the included studies was not performed, which limits the ability to assess the strength and reliability of the synthesized evidence. Given the heterogeneity and methodological limitations of the available literature, this is an important shortcoming that should be addressed in future meta-analyses. However, depending on the observed heterogeneity and potential bias in the included studies, appropriate statistical adjustments and cautious interpretation of the results were applied throughout the analysis.

## 5. Conclusions

After combining data from all recently published studies, we find that if EEA is performed, a strategy of GTR leads to a lower recurrence rate compared with STR plus radiation. This paper did not address the safety and complications of these two approaches. Ultimately, we support a maximal safe resection strategy that includes GTR, and propose that the prior literature has been biased by the inclusion of papers where a craniotomy was performed. Further prospective studies will be required to fully answer this question.

## Figures and Tables

**Table 1:** Summary of studies included in the meta-analysis assessing the link between GTR vs. STR with radiation and the risk of recurrence of craniopharyngioma after EEA resection.

Author and Year	Children/Adults	Follow-Up [Years]	Patients	Recurrence	Recurrence After GTR	Recurrence After STR and XRT
Bobeff, 2023	Mixed	4.8 (IQR 1.3–7)	95	16 (17%)	13%	38%
Apra, 2019	Adults	STR: 5.3 (2.1–9.9) GTR: 3.7 (1.7–9.9)	20	2 (10%)	0	33%
Madsen, 2019	Children	2	26	3 (12%)	8%	50%
Yamada, 2018	Children	7.8 (range 1.3–25.6)	61	7 (11%)	12%	0
Dho, 2018	Adults	2.6	63	2 (3%)	3%	0
Yano, 2017	Mixed	3.3 (IQR 1.7–5.8)	9	3 (33%)	29%	50%
Park, 2017	Mixed	2.9 (range 0.1–9.6)	72	8 (11%)	9%	17%
Patel, 2017	Children	4.7 (range 0.1–7.5)	15	2 (13%)	7%	100%
Turel, 2016	Adult	8.6 ± 7.1	26	7 (27%)	0	32%
Matsuo, 2014	NS	3.2 (range 0.5–6.5)	11	1 (9%)	0	100%
Koutourousiou, 2013	Mixed	3.2 (range 0.1–11.3)	33	9 (27%)	25%	33%
**TOTAL**	**Mixed**	**Mean/median 2–8.6**	**431**	**60 (14%)**	**10%**	**30%**

Abbreviations: EEA, endoscopic endonasal approach; GTR, gross total resection; IQR, interquartile range; STR, subtotal resection; XRT, radiation therapy.

**Table 2:** Summary of studies providing raw data, either included in the manuscript, Supplementary Materials, or available upon request, used for further survival analysis.

		GTR	STR Plus XRT
Children	N	41	17
	Follow-up [years]	2.8 (IQR: 0.9–5.0)	2.7 (IQR: 0.7–4.2)
	Recurrence	2/41 (4.9%)	7/17 (41.2%)
Adults	N	113	31
	Follow-up [years]	2.6 (IQR: 0.9–5.2)	3 (IQR: 1.2–5.5)
	Recurrence	13/113 (11.5%)	6/31 (19.4%)

Abbreviations: EEA, endoscopic endonasal approach; GTR, gross total resection; IQR, interquartile range; STR, subtotal resection; XRT, radiation therapy.

## Data Availability

No new data were created or analyzed in this study. Data sharing is not applicable to this article.

## Abbreviations

CI	confidence interval
CSF	cerebrospinal fluid
EEA	endoscopic endonasal approach
GTR	gross total resection
IQR	interquartile range
OR	odds ratio
P	progression
PRISMA	Preferred Reporting Items for Systematic Reviews and Meta-Analyses
QoL	quality of life
R	recurrence
XRT	radiation therapy
STR	subtotal resection
